# Barriers to Physical Activity Participation in Children and Adolescents with Autism Spectrum Disorder

**DOI:** 10.3390/healthcare12232420

**Published:** 2024-12-02

**Authors:** Sarvin Salar, Bojan M. Jorgić, Mihai Olanescu, Ilie Danut Popa

**Affiliations:** 1 Department of Sport Injuries and Corrective Exercises, Faculty of Physical Education, University of Guilan, Rasht 41996-13776, Iran; sarvin_salar@yahoo.com; 2 Faculty of Sport and Physical Education, University of Niš, 18000 Niš, Serbia; bojanjorgic@yahoo.com; 3 Faculty of Automotive, Mechatronics and Mechanical Engineering, Technical University of Cluj-Napoca, 400641 Cluj-Napoca, Romania; ilie.popa@mdm.utcluj.ro

**Keywords:** autism, questionnaire, barriers, factors

## Abstract

Background: Children and adolescents with autism spectrum disorders tend to participate in less physical activity. The purpose of this study was to identify barriers to physical activity participation in children and adolescents with autism spectrum disorder (ASD) based on parental reports. Methods: The sample comprised 370 children and adolescents with ASD, aged 8–20 years. Simple random sampling was selected. We designed a questionnaire and used the survey method for data collection. Data were analyzed using descriptive and inferential statistics. Results: The results revealed several barriers to physical activity participation: interpersonal barriers were the top priority, followed by friends and peers as well as psychological factors as the second and third priorities. Additionally, family, cognition, management and planning, social and cultural factors, and skills were identified as the fourth through eighth barriers, respectively. Conclusions: Understanding these barriers is crucial for developing effective school- and community-based strategies to promote physical activity participation.

## 1. Introduction

Autism spectrum disorder (ASD) is a neurodevelopmental condition characterized by various deficits in social skills, communication, sensory, neurological, and neuromotor functions, affecting approximately 1 in 36 children in the United States [1]. Individuals with ASD often experience delays in motor development and show a lack of engagement in physical activities [2]. Subjective assessments of physical activity levels indicate that children and adolescents with ASD are less likely to participate in physical activities compared to their typically developing peers [3]. Due to their insufficient participation in physical activities, children and adolescents with ASD are at a heightened risk of developing health-related issues, such as obesity [4]. Low levels of physical activity can increase the risk of chronic diseases and negatively impact the quality of life [5]. Studies have shown that individuals with ASD have a significantly higher likelihood of being overweight or obese compared to their typically developing peers [6]. Statistics indicate that children with ASD are 40% more likely to be overweight and obese compared to their typically developing peers [7]. There is a well-documented positive association between physical activity and health, and the importance of promoting physical activity among children and adolescents has been increasingly recognized [8]. The WHO guidelines recommend that children and adolescents engage in moderate-to-vigorous physical activity (MVPA) for 60 min per day (mostly aerobic physical activity). They also recommend that vigorous physical activities and muscle and bone strengthening activities should each be incorporated at least 3 days per week [9].

This new guideline is similar to physical activity guidelines from most countries (e.g., Canada [10], Australia [11], USA [12]).

Studies have shown the positive effects of PA on cognition, behavior, and motor skill [13] and improvements in physical fitness, psychological function, and quality of life in individuals with autism [14].

However, children and adolescents with ASD often face limited opportunities for physical activity and encounter challenges due to difficulties in social interactions and motor skills [15], as well as impairments in motor, social communication, sensory, and behavioral domains [16]. Some studies have identified barriers to physical activity in children with ASD. These barriers include low levels of motivation, low interest in physical activity [17,18], and preferences for simple, sedentary activities [16]. Other barriers are related to the characteristics of ASD, such as impaired communication, limited social interaction, and motor difficulties [19]. Parental support and planning are crucial indicators of participation in physical activity among children with disabilities, as they often depend on their parents for all aspects of engagement [20]. Researchers have highlighted the importance of parents as key collaborators in promoting physical activity for their children [21] and reported that parents face both motivations and barriers in involving their children in physical activity [22]. 

The importance of parents in not only in prompting physical activity but also acquiring motor skills could facilitate the adherence to and maintenance of physical activity throughout life. For example, if these children have learned to cycle, it will motivate them to be more active and try other sporting activities more easily. There are studies that show that children with autism who learn to cycle are more physically active and have a better physical condition and body composition than their peers who have not learned. Learning an age-appropriate motor behavior, such as riding a two-wheeled bicycle, is helpful in establishing an environment to enhance social skill generalization and peer and family relationships. The generalization of social skills includes more independence, confidence, communication, and coping. Parents indicated that obtaining the ability to ride a two-wheeled bicycle aided in creating an environment for family activities without making special accommodations for their youth with ASD [23]. Parents play a significant role in creating participation opportunities for their children through specific strategies and decision-making processes [24]. 

Despite the growing attention to physical activity participation for individuals with autism [14], the limited participation in physical activity shown among children and adolescents with ASD in Iran and barriers to participation have received less focus [25], while persistent barriers hinder participation, especially as individuals age [26]. Therefore, there is also a need for research that comprehensively describes the barriers to physical activity participation in children and adolescents with ASD. Our study aims to investigate these barriers based on parental reports for Iranian children and adolescents with autism spectrum disorders.

## 2. Materials and Methods

This study is descriptive and cross-sectional in nature. We developed a custom questionnaire and employed the survey method for data collection.

### 2.1. Participants

This study’s statistical population consisted of 370 children and adolescents, aged 8–20 years, (12.27 ± 3.2), with a clinical diagnosis of autism spectrum disorders according to the Diagnostic and Statistical Manual of Mental Disorders, 5th Edition (DSM-5), from Iran.

The statistical samples of the present study include 370 children and adolescents with autism disorder from Iran (both genders), aged 8–20 years. Simple random sampling was selected. The number of statistical samples was considered to be 384 according to Morgan’s table and Cochran’s formula [27]. 

In total, 370 questionnaires were received and analyzed. This group included 233 boys and 137 girls. Participants were selected through random sampling. In this selection method, all the individuals have an equal opportunity to participate in the study, where the selection process is entirely based on luck [28].

In this study, the parents completed the questionnaires. We identified the parents through their children’s records from autism associations across Iran. We communicated the study’s main purpose, importance, and necessity to the directors and managers of these associations and asked them to share a summary of this information with parents to gauge their interest in participating. Following this, the directors introduced us to parents who were willing to participate in the research. As a result, we were able to recruit 400 parents of children with ASD. They were recruited through various disability-related organizations and communities based on our inclusion criteria and their interest in the study. In the next step, we obtained informed consent from the participants, and 400 parents received the questionnaire electronically via email and social media. We asked parents to carefully read each question and respond based on their perceptions of their children’s experiences. They were encouraged to reach out to the lead author with any questions or concerns using the provided contact number and email address. Parents completed the questionnaire and submitted their responses electronically. We sent three reminders, two weeks apart, until data saturation was achieved. After three months, we collected all the completed questionnaires and began the analysis using SPSS23 and Smart PLS3 software. We found that 370 of the questionnaires were fully completed and included them in the analysis.

### 2.2. Measurement Instrument

In this study, we utilized a researcher-made questionnaire to investigate barriers to physical activity participation among children and adolescents with autism spectrum disorders. The questionnaire, identified as a reliable tool for measuring these barriers, demonstrated a Cronbach’s alpha coefficient of 0.985. The creation of the questionnaire followed several steps. Initially, we conducted exploratory research by reviewing relevant literature, including national and international articles, over two months. From this review, we extracted data that we deemed important and likely to negatively impact physical activity levels in children and adolescents with ASD. The research team collaborated to design the questionnaire, which included 68 items across 8 components, based on a 5-point Likert scale ranging from very satisfied to very dissatisfied. In the next step, we formed a panel of 14 experts, including four specialists in adapted physical education, four in motor development in autism, four in physical activity and physical education, one child psychiatrist specializing in autism, and one expert in rehabilitation. All panel members had research and field experience working with children. We requested their quantitative opinions on the questionnaire’s content, alignment with research objectives, number of items, and clarity. Additionally, we sought their qualitative feedback on each question based on a 3-point Likert scale: “be related”, “be necessity”, and “be important”. We analyzed their responses quantitatively using SPSS23 and qualitatively by checking the appropriateness of the questions and merging or deleting some as needed. The finalized questionnaire was then distributed to 400 parents, who were asked to rate their knowledge and perceptions of their children’s physical activity participation. We collected a total of 370 completed questionnaires. These were evaluated, and their reliability was analyzed using SPSS23 and SmartPLS3 software.

### 2.3. Statistical Methods

To evaluate the fit of the measurement model in the initial stage, we examined factor loadings and significance coefficients and assessed reliability, convergent validity, and divergent validity.

We assessed the Content Validity Index (CVI), which is the degree to which an instrument has an appropriate sample of items for the construct being measured based on experts’ ratings of item relevance. Also, we assessed the CVR (content validity ratio), which is a linear transformation of a proportional level of agreement on how a scale with excellent content validity should be composed of 0.78 or higher, and our CVI and CVR scales were higher than 0.78 and acceptable [29]. 

**Figure 1:**
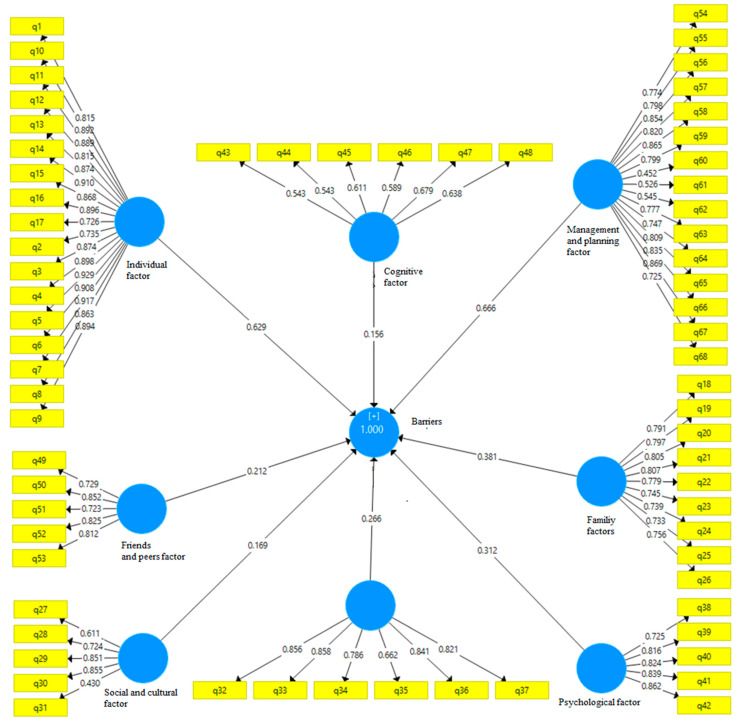
Structural equation modeling.

**Figure 2:**
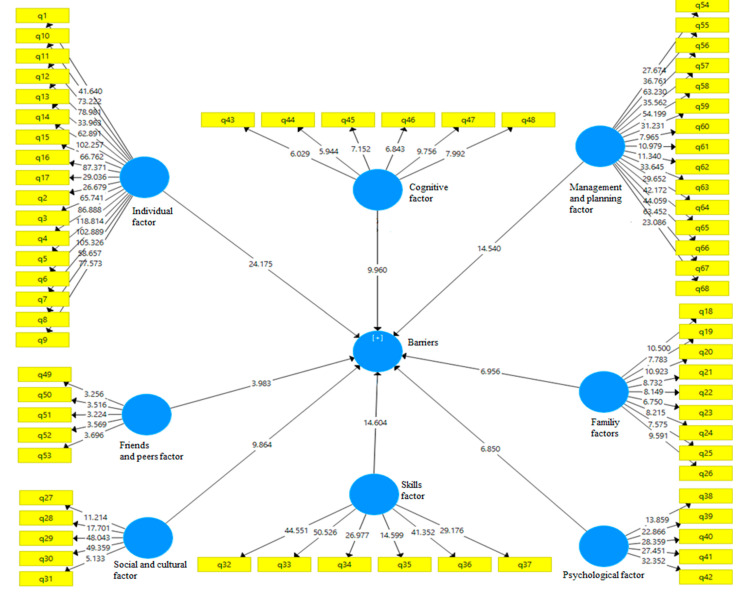
Structural equation modeling.

Model reliability was measured using composite reliability and Cronbach’s alpha. Convergent validity was assessed through the average variance extracted (AVE) index, which indicates the degree of correlation between a construct and its indicators—the higher the correlation, the better the fit. The reliability and validity of the questionnaire (all items) were verified through confirmatory factor analysis, yielding values above 0.7, thus confirming the questionnaire’s reliability. Initially, the data were run through the measurement model to check if all questionnaire items had acceptable factor loadings. The results showed that the standardized regression weights for all items exceeded 0.70, and the composite reliabilities were above 0.80. These findings supported the assumptions of internal consistency and reliability of the measurement model. We assessed the convergent validity of the constructs to determine if measures that should theoretically be related are indeed related. Convergent validity was evaluated using the average variance extracted (AVE). The AVE for all constructs was equal to or greater than 0.50, confirming that convergent validity had been achieved for the measurement model [30]. Additionally, the significance coefficients for all structures exceeded 1.96, and the variance inflation factor was less than 5, indicating a good model fit. To standardize the model, the Standardized Root Mean Squared Residual (SRMR) index was used, and the obtained value of 0.056, being less than 0.080, indicated a good fit. Consequently, the data analysis results showed that the model was well-fitted to the data, allowing us to test the hypothesis and examine the relationships between the independent and dependent variables Figure 1 and Figure 2.

Exploratory Factor Analysis (EFA) was used, which is a method within factor analysis whose overarching goal is to identify the underlying relationships between measured variables. The number of factors to retain was determined by the Kaiser criterium. The Kaiser–Meyer–Olkin test [31] is a statistical test used in factor analysis to determine if the data are suitable for factor analysis. The KMO is calculated based on the correlation between the variables. It ranges from 0 to 1, with values closer to 1 suggesting the variables are correlated and the data are well-suited for factor analysis; otherwise, the variables are uncorrelated and there may not be a common factor influencing them [32]. Bartlett’s test [33] was used to test if k samples have equal variances. In this study, KMO = 0.918 and (χ^2^ = 22,989/185, Sig: 0.001). They were statistically significant.

The questionnaire consisted of 68 items, with variables related to barriers to physical activity in children and adolescents with ASD covering 8 areas: individual, family, social and cultural factors, skill factors, friends and peers, psychological factors, cognition factors, and management and planning. One-sample *t*-tests were conducted to determine the role of individual (T = 22.666), family (T = 9.246), friend and peer (T = 10.976), psychological (T = 3.921), cognitive (T = 11.149), social and cultural (T = 4.90), skill (T = 9.41), and management and planning factors (T = 2.192) in participating in physical activity. The mean ranking Friedman test was used to rank the factors that represent barriers to participating in physical activity.

## 3. Results

The participants’ demographic data are indicated in Table 1.

Initially, a one-sample *t*-test was conducted to determine the role of interpersonal, family, friend and peer, psychological, cognitive, social and cultural, skill, and management and planning factors in participating in physical activity (shown in Table 2). One-sample *t*-tests were used to determine the role of the variables in participation in PA.

The questionnaire includes factors such as the individual, psychological factors, the family, cognition, social and cultural factors, skills, friends and peers, and management and planning. Individual factor include a lack of time, a lack of energy, the fear of injury, feeling pain or exhaustion, a lack of knowledge about how do exercise and being active and utilizing exercise tools, the failure to modify tools and environments, a lack of interest, unpleasant experiences, and a lack of reason. Family factors include family concerns, a ack of knowledge, expensive costs, a lack of support and encouragement, family cultures and beliefs about physical activity, and the non-participation of parents in physical activities. Social and cultural factors include people’s ignorance, wrong judgment and negative attitudes to autism, social norms, being ashamed of appearance and abilities, and the fear of unsafe place and being assaulted. Skills factors include adequate motor skills and physical fitness, poor motor coordination, a lack of balance, and the fear of falling. The friend and peer factors include not being accepted by friends and peers or being rejected by them, a lack of encouragement, understanding and insufficient support from friends and peers and bullying, the absence of another person with a similar disability by one’s side, and the absence of another person with a similar disability next to the child. Management and planning factors include the failure to receive the necessary support and sufficient support from related institutions and organizations, a lack of special sports facilities for children and adolescents with autism, the failure to receive the necessary support and sufficient support from relevant institutions and organizations, a lack of up-to-date technologies and standard equipment, the lack of trained adapted physical education professionals and their lack of knowledge about the needs of children on the autism spectrum, a lack of planning for the implementation of adapted physical activity programs, a lack of opportunities for social participation, a lack of family recreational sports programs, the negative attitude of employees and their unwillingness to work with people with autism, programs focusing on team and competitive sports instead of non-competitive physical activities, a lack of access to transportation services, the laws and regulations of society regarding disability rights, and a lack of available media information about physical activity in people with autism spectrum disorder. Cognition factors include the unpredictable behavioral characteristics of autism, co-occurring secondary diseases and disorders, cognitive disorders, and learning and educational problems. Psychological factors include the fear or anxiety caused by exposure to new environments and resistance to accepting changes in one’s daily routine, a lack of self-confidence and self-esteem, sleep disorders, and depression. For each question, a 5-point Likert scale was offered, which was five different options including two extreme, two intermediate, and one neutral opinion. Parents were asked to say how much they agreed with each phrase. For example, a “lack of expert adapted physical education teachers is such a barrier to participation in physical activity”. Respondents would choose from five options ranging from “strongly agree” to “strongly disagree”.

As shown in Table 1, the average responses to questions related to all these factors were higher than the theoretical average (3), and these differences were statistically significant (*p* < 0.01). Therefore, all these factors are perceived as barriers to participation in physical activity.

In the next step, we prioritized the identified barriers using the mean ranking Friedman test (Table 3, which shows the mean ranking Friedman test). 

The results indicated that the “individual” factor, with an average rank of 6.17, was considered the most important barrier, while the “skill” factor, with an average rank of 3.13, was deemed the least important. Additionally, the results of the one-sample *t*-test at *p* ≤ 0.05 showed that for all variables, the significance level was higher than 0.05, indicating no significant difference between girls and boys in the barriers to participation in physical activity.

We also prioritized the subset factors of each variable by using the Friedman test. In the interpersonal variable, we identified that the statement “The child does not see any reason to do physical activity and does not think it is beneficial” had an average importance of 10.40, making it the most significant factor. Conversely, “The child’s inability to navigate unfamiliar environments” was the least important individual factor, with an average of 4.99. For the family variable, the statement “Parents prefer their children to do school assignments rather than participate in physical activity” had the highest importance, with an average of 5.18, while “Physical activity takes too much time from other family activities” was the least important, with an average of 4.77. In the social and culture variable, the statement “The child is embarrassed about how they look when doing physical activity with others” was the most critical factor with an average of 3.51, whereas “Unsafe environment and fear of assault” was the least important with an average of 2.77. Regarding the skill variable, “Weakness of physical fitness” was the most crucial factor, averaging 4.36, and “Lack of sufficient motor coordination” was the least important, averaging 2.86. In the psychological variable, “Urinary incontinence” was the most significant factor, with an average of 3.44, while “Stress due to exposure to new environments and resistance to change” was the least important, with an average of 2.57. For the cognition variable, “Cognitive disorders” had the highest importance, with an average of 3.74, and “Unpredictable behavioral characteristics” was the least important, with an average of 3.25. In the friend and peer factor, “Not having a partner with a similar disability to participate in physical activity with” was the most important factor, averaging 3.13, while “Bullying by friends and peers” was the least important, with an average of 2.54. Finally, in the management and planning variable, “Lack of consideration, attention, and support by physical activity organizations” was the most critical factor, with an average of 8.86, whereas “Focus on competitive physical activity and sports instead of enjoyable physical activity” was the least important, with an average of 6.62.

## 4. Discussion

The purpose of this research was to determine barriers to participation in PA in children and adolescents with ASD based on parent reports. The results of this study revealed eight factors that limit participation in physical activity among children and adolescents with ASD in Iran. Understanding these barriers, as mentioned in the previous section, is crucial. Some studies have shown that children and adolescents with autism spectrum disorders are less likely to engage in physical activity than their typically developing peers. Limited participation in physical activity plays a significant role in increasing the risk of overweight and obesity in children and adolescents with ASD, thereby raising their risk of chronic health-related diseases and negatively impacting their quality of life [1].

Movement/sport training can help improve fundamental motor skills, physical fitness, motor outcomes, psychological function, and quality of life in children and youth with autism [14]. The positive effects of PA on cognition, behavior, and stereotypic/repetitive behaviors in youth with ASD have also been shown [26].

A study in Iran indicated that only 12% of Iranian children with ASD were physically active. Children were predominantly engaged in solitary play rather than social play activities. Gender, family income, and household structure were found to be associated with activity scores. Financial burden and a lack of opportunities were noted as the leading barriers to physical activities. The findings indicated a low rate of physical activity participation in children with ASD that is closely associated with sociodemographic variables [34].

Stanish et al. [35] reported that adolescents with ASD spend more time in sedentary behavior and less time in physical activity than their peers without disabilities. Furthermore, Stanish et al. [35] reported that most participants with ASD enjoyed participating in physical activity and were aware of its benefits. However, they often expressed that they were too busy for physical activity, found it boring, or feared getting hurt. Healy [6] identified three key themes: individual challenges related to physical ability, sensory issues (such as auditory, heat, and tactile sensitivity), and a fear of injury that seemed to limit the quality of participation in physical education for children with ASD. An important factor in understanding the facilitators and barriers to physical activity is the influence of family members, especially parents and siblings, as children heavily rely on these individuals for everyday activities [36]. The results of our study indicated that parents from diverse backgrounds generally had positive beliefs about the benefits of physical activity for their children with ASD, which is consistent with the findings of Obrusnikova [37]. In this study, within the family factor, parents reported that despite recognizing the benefits of physical activity (PA), they lacked the knowledge to support their children’s participation and did not know how to encourage them. This finding is similar to those of Must et al. [38], who identified parents’ lack of skills to promote their child’s physical activity as a key barrier. Parents of children with disabilities value PA and recognize its psychological and health benefits, believing that their child’s participation in PA is associated with these values [22]. In our study, parents reported that their children’s sedentary behavior and lack of regular physical activity might limit their participation in PA. Furthermore, parents expressed that their high expectations for their child’s success in PA might also act as a barrier to participation.

In the intrapersonal factor, parents identified several barriers to physical activity (PA) participation for their children, including a lack of time, the fear of injury, pain, physical discomfort, boredom, a lack of understanding and adherence to rules, unpleasant past experiences with PA, dependence on parents, the need for parental supervision, the requirement for environmental adjustments, and a lack of knowledge about how to participate in PA and use sports equipment. Among the interpersonal barriers, the statements “parents do not have time and energy” and a lack of peer partners for exercise were the most significant barriers, respectively. To address the lack of motivation for PA, parents emphasized that their children with ASD should perceive PA as important, enjoyable, interesting, useful, and easy to perform [39]. Additionally, parents perceived several barriers for their children with developmental disabilities in PA participation, such as family priorities, a lack of available opportunities in their environment, the need to modify activities, and the need for specialized support. Parents often chose activities that did not present extensive social and physical demands for their children, leading to increased participation in sedentary and solitary activities [40]. Children and adolescents with ASD often lack someone to do physical activity with and do not have peer exercise partners [37]. Moreover, parents reported that establishing predictability through schedules and routines and allowing their children with ASD to have choices during PA provided these children with a sense of control over each situation, ultimately increasing their PA participation [41]. 

In our study, the lack of friends and peers as partners in physical activity (PA), rejection and exclusion by friends and peers, bullying, and insufficient support and encouragement from friends and peers were identified as key barriers in the friend and peer factor. The absence of peer exercise partners was one of the most significant barriers [39]. Healy [6] found that peer interactions, relationships, and experiences with peers were crucial factors influencing the quality of participation in physical education. Children with ASD had both positive and negative experiences in physical education, enjoying time with peers and friends but also experiencing bullying and feelings of exclusion. Additionally, the lack of friends and peer support [37] and having few friends to engage in physical activity with [30] are consistent with our findings. Must et al. [38] reported that 60% of parents of children with ASD stated that their child required too much supervision, limiting their participation in PA, which aligns with our study. Social challenges, motor skill deficiencies, and sensory hypersensitivity can make childhood sports challenging, leading to negative experiences and reduced motivation for children with ASD to participate in PA [42]. Our study also highlighted that parents reported sensory organization disorders as barriers. They noted that sweating and itchy skin following motor activity or engaging in PA was bothersome for their children and that the light and noise in some environments were unsuitable. Ginis [43] and Must [38] identified perceived barriers including a lack of energy, lack of motivation, and preference for other activities, which align with our findings.

In the study by Obrusnikova [37], the most significant intrapersonal barriers were identified as a lack of interest and motivation for physical activity (PA) as well as a lack of confidence in their ability to participate. Similarly, Must et al. [38] also reported a lack of interest and motivation as barriers. These findings align with our results, where parents reported that inadequate motivation and a lack of self-confidence in their abilities are perceived barriers to PA participation. Financial constraints are widely recognized as major factors limiting participation in PA [38,43,44]. Our study confirmed this, as parents cited the cost and lack of insurance coverage for physical activity services as perceived barriers. Additionally, deficits in social interactions can limit opportunities for participation in physical activities with peers [45]. This is consistent with our findings, where parents reported a lack of sufficient social interaction as a barrier to PA participation. Our study also showed that societal judgments, stigmatization, and negative attitudes towards autism, along with societal expectations, are significant barriers. Motor skill delays may result in challenges related to PA participation, such as low motivation, poor gait and posture, and a lack of rhythm [46]. The lack of sufficient motor skills was also reported by Shields [44], which aligns with our findings. Parents in our study reported that insufficient motor skills, physical fitness, motor coordination, difficulty in learning movement skills, weakness in balance, and abnormal gait patterns are key barriers that limit PA participation in Iranian children and adolescents with ASD. Physical activity is considered critical for promoting motor skills in children without ASD, and there is an association between PA and motor skills in young children with ASD. KU [47] found a bi-directional relationship between these factors, noting that while children with ASD can develop motor skills, they participate in less PA than their peers. Having an appropriate level of motor skills is a prerequisite for PA participation, but children with ASD experience motor skill deficits that limit their participation.

Additionally, disordered sleep, psychopharmacological medication, atypical eating patterns, metabolic abnormalities, social anxiety, environmental barriers, and other disabling characteristics associated with autism were reported by Srinivasan et al. [16]. Duquette et al. [48] found that physical and social barriers impact young adults with ASD in their participation in physical activity. Our results also highlighted management and planning issues, such as a lack of social support; inadequate sports facilities or facilities specifically designed or modified for children and adolescents with autism; outdated technologies and standard equipment; insufficient assistance, support, and supervision from sports facility staff; and a lack of knowledgeable physical educators or adapted physical educators for children and adolescents with ASD. Additionally, parents reported a lack of community-based programs, recreation and leisure time physical activity opportunities, negative attitudes from staff, staff unwillingness to work with children with autism, difficulties accessing transportation services, and a lack of transportation. These findings are consistent with another study [37]. Buchanan [49] explored the importance of intrapersonal factors, interpersonal relationships, and community factors in keeping individuals with ASD engaged in physical activity. Families can take advantage of community settings or activities with other individuals to encourage participation in PA [49]. The lack of qualified professionals to provide appropriate PA services for children with disabilities was also highlighted in a study by Columna [22], which aligns with our findings. Parents emphasized the need for more programs and qualified professionals and called for greater collaboration and communication with PA professionals [22]. Additional barriers that were identified included ignoring the laws and rights of people with disabilities in the community, a lack of media awareness about the PA of people with autism, and limited access to school-based opportunities. A lack of play opportunities and physical activity programs to join were also reported by Memari et al. [34] and Obrusnikova [37]. In Obrusnikova’s study [37], the most important community barriers were the lack of appropriate and affordable PA opportunities, staff training, and community safety, which is in line with our findings.

Physical activities can aid in the development of social skills, and it is possible that individuals with autism can develop these skills in a developmental activity environment.

By teaching children how to interact in a physical activity environment, they can improve their physical activity levels and positively impact their health in various ways. Investigating climatic, cultural, and social factors is crucial for understanding physical activity patterns [50]. A study suggests that teachers and educators are required to engage children and adolescents with autism in a variety of movement experiences that include games and motor skills and physical fitness. It is important for them to be aware of using strategies in this field. Before inviting a person to participate in physical activities, it is recommended that teachers and trainers explain their expectations and what the person should do so that the autistic child and adolescent understands the purpose of the activity [51]. Studies emphasize the importance of non-competitive physical activity experiences in a friendly environment with flexible social interactions because competition has been reported as an important factor in giving up physical activity from the perspective of parents. Studies suggest that having people for physical support during physical activities can be effective. Therefore, physical activity opportunities should be accessible and non-competitive [52]. Providing opportunities to interact with peers is considered one of the positive aspects of promoting participation in physical activities. For example, spaces with very low and high light, narrow and long corridors, crowded and noisy environment, and very small rooms for children and adolescents with autism spectrum disorder create anxiety and fear. Anticipating barriers and formulating appropriate strategies can contribute to a rich and inclusive experience for people with autism spectrum disorder. According to this study, every student with autism spectrum disorder has their own experiences, emotional feelings, and barriers that educators and teachers must be aware of. The awareness of these obstacles can start with a two-way conversation and lead to planning [53].

The development of community-based physical activity programs for children and adolescents with autism should be considered. Specific suggestions for parents, schools, or public policies to promote physical participation based on the results will be useful.

### Strengths and Limitations

The limitations of this study include the following. One limitation is the limited evidence on how interfering factors over time might influence PA participation among children and adolescents with ASD. Our study relied on parents’ reports, so we recommend future research to also consider the perspectives of children and adolescents themselves to provide additional insights. Factors affecting PA participation in children and adolescents with ASD can vary significantly depending on different contexts and the severity of the disorder. The bias introduced by using the self-report by parents might be one of our research limitations.

Moreover, since our sample was drawn exclusively from Iran, the findings may not be generalizable to other populations for the whole world. Although most of these barriers can be similar, the specific cultural context of each country can be highlighted.

Therefore, further research is needed to examine differences in PA participation based on gender, subject matter, and other factors such as the severity of autism and the presence of comorbid disorders. We also suggest that future research identifies PA barriers in typically developing peers and compares these with the barriers faced by children and adolescents with ASD. Despite these limitations, the strengths of this study include its unique focus on Iranian children and adolescents with ASD and the use of standardized, reliable survey measures. More evidence is needed to fully understand the challenges and barriers to PA participation for children and adolescents with ASD.

## 5. Conclusions

The present research is one of the first comprehensive studies to investigate the barriers influencing PA participation among children and adolescents with ASD in Iran, based on parents’ perceptions. The factors highlighted by parents provide valuable insights for teachers and can support the successful inclusion of students with ASD in Iran. Understanding these barriers comprehensively can lead to more detailed programming aimed at overcoming them and promoting PA participation. Professionals and adapted physical educators can play a crucial role in educating parents about appropriate physical activities and their benefits. Also, they need to consider and be particularly supportive of parental priorities and schedules. These findings emphasize the importance of community-based PA programs that meet the special requirements of this population and advocate for policies that encourage inclusion strategies in schools and other government-supported organizations. Addressing the identified research barriers will better inform guideline recommendations in children and adolescents, and future work should aim to prioritize decreasing these barriers. In the meantime, investment and leadership is needed to scale up known effective policies and programs aimed at increasing activity in children and adolescents. Also, we suggest that trainers and adapted physical educators’ teachers improve their knowledge and employ approaches based on the needs and abilities of children and adolescents with autism spectrum disorder in schools and community settings.

## Figures and Tables

**Table 1:** Participants’ demographic data.

Participants Demographic Data	
Sex	63% (*n* = 233) boys
	37% (*n* = 137) girls
Age	Participants’ ages: 8–20 years (mean age: 12.8 ± 3.75 years)
	Parents’ ages: 25 to 50 years (mean age: 38.4 ± 5.10 years)
Educational levels	61% elementary school education (*n* = 226)
	23.8% junior high school (*n* = 88)
	13.5% high school (*n* = 50)
	1.1% preschool education (*n* = 4)
Severity of autism	31.8% mild autism (*n* = 87)
	3.3% moderate autism (3.3% had moderate autism (*n* = 138))
	24.9% severe autism (*n* = 88)
Type of comorbid disorders	15.1% anxiety disorders
	17.3% learning disability
	19.5% attention deficit hyperactivity disorder (ADHD)
	16.5% intellectual disability
	23.2% developmental coordination disorder
	4.1% all items as a comorbid disorder
	3.5% without any comorbid disorder
	0.8% other options

**Table 2:** One-sample *t*-tests to determine the role of the variables in participation in PA.

Variable	Mean	Standard Deviation	*t* Value	*p*
individual factor	4.14	0.97	22.666	0.001
family factor	3.52	1.09	9.246	0.001
social and cultural factor	3.17	0.70	4.90	0.001
skills factor	3.51	1.06	9.41	0.001
psychological factor	3.76	1.02	3.921	0.001
cognitive factor	3.46	0.79	11.149	0.001
friends and peers	3.61	1.07	10.976	0.001
management and planning	3.11	1.04	2.192	0.029

(*p* < 0.05).

**Table 3:** Mean ranking Friedman test.

Variable	Rank	Mean Rank	Friedman Test	*p*
individual factor	1	6.17	368.409	0.001
family factor	4	4.59		
social and cultural factor	7	3.84		
skills factor	3	4.86		
psychological factor	8	3.13		
cognition factor	5	4.51		
friends and peers	2	5.02		
management and planning	6	3.88		

## Data Availability

Data are available on request due to restrictions, e.g., privacy or ethics. The data presented in this study are available upon request from the corresponding author. The data are not publicly available to protect the privacy of the participants in this research.
